# Efficacy of a neuroscience informed psychoeducation intervention on cognitive, emotional, and substance use outcomes in college students: a pilot study

**DOI:** 10.3389/fpsyt.2025.1655909

**Published:** 2025-09-18

**Authors:** Tara Rezapour, Kayla L. McLean, Elena Psederska, Swara Chokshi, Khashayar Niki Maleki, Hamed Ekhtiari, Jasmin Vassileva

**Affiliations:** ^1^ Institute for Drug and Alcohol Studies, Virginia Commonwealth University, Richmond, VA, United States; ^2^ Department of Cognitive Psychology, Institute for Cognitive Science Studies (ICSS), Tehran, Iran; ^3^ Department of Cognitive Science and Psychology, New Bulgarian University, Sofia, Bulgaria; ^4^ Metacognium Limited Liability Company (LLC), Irvine, CA, United States; ^5^ Department of Psychiatry and Behavioral Sciences, University of Minnesota, Minneapolis, MN, United States; ^6^ Department of Psychiatry, Virginia Commonwealth University, Richmond, VA, United States

**Keywords:** neuroscience, psychoeducation, substance use, prevention, metacognitive

## Abstract

**Introduction:**

Despite revolutionary advances in understanding the neurobiology of substance use, these insights have not been translated into effective prevention or intervention programs. To address this gap, we developed *Neuroscience-Informed Psychoeducation for Addiction* (NIPA), a mobile app designed to enhance metacognitive awareness, increase cognitive resilience, and promote neurocognitive skills for stress coping and substance misuse prevention. NIPA targets key cognitive functions—attention, memory, cognitive flexibility, and decision-making —by integrating neuroscience-based psychoeducation with gamified neurocognitive tasks and metacognitive training to enhance engagement and real-life application.

**Objective:**

In this study, we aimed to examine whether using a neuroscience-based approach could change young adults’ attitudes and intentions to use drugs and alcohol, and improve their executive functions, emotional health, and decision-making.

**Method:**

Sixty-eight undergraduates from a large urban public university participated in this pilot study. Eligibility criteria included: age ≥18, prior alcohol/tobacco/cannabis/other drug use, and ability to download and complete the app. Prior to the intervention, participants completed self-report cognitive, emotional, and substance use questionnaires; these were repeated after completing four 20-minute-long NIPA sessions. NIPA incorporates metacognitive training and game-based neurocognitive tasks delivered through cartoons, animations, and videos to increase awareness about the effects of drugs and alcohol on brain and cognition. Pre–post intervention changes were analyzed using Wilcoxon signed-rank and binomial tests.

**Results:**

Findings reveal significant reductions from pre- to post-intervention assessment in self-reported deficits in executive function (Z=-7.11; *p*<0.001) and emotional distress including depression (Z=-2.58; *p*=0.010) and anxiety (Z=-2.49; *p*=0.013), and an increase in metacognitive awareness (Z=-3.07; *p*=0.002). Additionally, assessment of decision-making revealed reduced delay discounting of large magnitude rewards (Z=-2.11; *p*=0.035) and increased probability discounting of small probabilities (Z=-3.177; *p*=0.001), indicating increased sensitivity to uncertainty. Finally, participants reported significantly lower intentions to use and lower actual use of nicotine and cannabis, and lower binge drinking from pre- to post-intervention assessment.

**Conclusion:**

These preliminary results support the potential of NIPA as an effective tool for increasing metacognitive awareness and enhancing cognitive resilience against stress and uncertainty. Future studies with larger samples, including a control group and follow-up assessments, are required to support these findings and assess the long-term effects of the intervention.

## Introduction

The use of illicit drugs among adolescents remains a persistent concern worldwide. Despite declining rates of adolescent substance use in the latest Monitoring the Future (MTF) study, new concerns emerge about recent trends, particularly since the COVID-19 pandemic (1). For instance, a new analysis of Centers for Disease Control and Prevention (CDC) data from 2010 to 2021 shows a dramatic rise in overdose deaths among teens, primarily attributed to illicit fentanyl use. Additionally, 30.7% of 12^th^ graders reported using cannabis in the past year (2). E-cigarette use has gained widespread popularity among youth, with an estimated 2.55 million U.S. students reporting use (3, 4). With regards to alcohol use, 5.6 million youth between 12 to 20 years of age reported drinking alcohol beyond “just a few sips” in the past month and 3.3 million reported binge drinking at least once in the past month (5). Although both alcohol and e-cigarettes are legal substances, there is ample evidence supporting a progression in substance use, beginning with legal substances and advancing to cannabis and illicit drugs such as heroin, other opioids, methamphetamine, or cocaine (6, 7). Given the significance of these alarming data on the new wave of illicit drug use among adolescents, and the association between substance use, criminal behaviors, and mental health problems, it is crucial to develop effective preventive interventions for adolescents (8).

Contemporary perspectives on substance use vulnerability are closely tied to neurocognitive factors, some of which are considered as precursors of substance use in adolescence. Findings from neuroimaging and neuropsychological studies suggest compromised functioning of certain cognitive processes, such as inhibitory control, attention, cognitive flexibility, and working memory, as well as structural and functional abnormalities in specific brain regions, including the prefrontal cortex and the limbic system (9). Poor neurobehavioral performance and disrupted brain activation during impulse control, reward processing, and working memory tasks have been linked to substance use in adolescents (10). This neurocognitive vulnerability has been explained by a group of theories classically known as the “dual systems model” (11, 12) or the “maturational imbalance model” (13). According to these models, risk-taking in adolescents is the result of imbalanced maturation between different brain networks. These include the prefrontal and parietal cortex, which are involved in deliberative, planful, and goal-directed behavior (System 2), and regions such as the ventral striatum and ventromedial prefrontal cortex, which are involved in reward and affective processes (System 1) (12, 13). The dual systems model posits that System 1 undergoes a rapid development, leading to an increased reward sensitivity and risk-taking during adolescence (14), while the prolonged maturation of brain regions involved in System 2 results in immature impulse control and reduced motivation for goal-directed behaviors (15).

In light of the growing interest in studying neurocognitive markers of substance use vulnerability, recent prevention approaches have been increasingly influenced by knowledge about the brain and neuroscience. One strategy used in this approach is to increase individual’s awareness about the structures and functions of the brain and how they change with chronic drug use (16). This form of self-awareness, referred to as metacognition, is described as knowledge of one’s own cognitive processes and the use of this knowledge to regulate cognition (17, 18). Individuals with intact metacognition can monitor their cognitive functions, recognize when challenges arise, and acknowledge the need to take action to address these issues or become more conscious of their brain health and adaptability skills. In this regard, a specific form of psychoeducation intervention has been developed, referred to as “neuroscience-informed psychoeducation (NIP)”, which places greater emphasis on neuroscience and brain literacy. By this novel approach, people could better relate their own behaviors to specific neurocognitive processes without fear of being stigmatized. Due to the non-stigmatizing nature of the NIP, individuals may benefit more from neuroeducation, as they are often more interested in the biological basis of mental health problems, place greater credibility and trust in the information, and feel increased compassion and empathy for themselves. More importantly, because NIP provides neuroscientific knowledge without advising or blaming individuals, it is particularly well-suited for addressing problems in adolescents (19).

To our knowledge, this approach has been applied in three harm reduction prevention programs for substance use so far. The first is the *The Illicit Project*, developed by Debenham and colleagues, which focuses on educating adolescents about the effects of certain substances on brain development and neuroplasticity (20). The second program is the *Just Say Know* prevention program, which has been shown to influence adolescents’ knowledge and attitudes toward drug and alcohol use (21). This program explains how brain structure and function can be changed by drugs and alcohol, leading to risky behaviors and continued substance use. The third neuroscience-based program is *NIPA (Neuroscience-Informed Psychoeducation for Addiction)*, recently developed by our team, which incorporates similar principles (22). Preliminary results support the acceptability and effectiveness of such interventions in increasing adolescents’ metacognitive awareness and improving mental health, as well as in reducing drug and alcohol consumption. In light of the potential utility of NIP for adolescents, integrating this approach into preventive interventions that explain complex neurocognitive mechanisms in an accessible and relatable manner, could enhance adolescents’ metacognitive awareness and their ability to control their behaviors based on neuroscientific knowledge and insight.

In addition, recent advances in eHealth technologies, such as websites, mobile apps, and wearable devices, have created numerous opportunities to deliver mental health interventions as scalable, cost-effective, and widely accessible services. These platforms allow individuals, particularly those who may avoid in-person sessions due to stigma, fear of being judged, or lack of financial resources, to receive therapeutic interventions and share their problems anonymously. Additionally, these technologies enable developers to provide educational content using various modes of presentation, such as verbal and visual, to enhance the learning experience in accordance with pedagogical practices (23). Therefore, delivering NIP through digital platforms for adolescents could also have added value in terms of engaging them more effectively in the learning process.

To this end, NIPA has been developed as a novel digital tool designed to improve adolescents’ metacognitive awareness and enhance their resilience against substance use, delivered through an interactive mobile app. In this study, we aimed to investigate the effect of NIPA on cognitive and emotional processes related to decision-making and mental health, as well as drug and alcohol use, motivation, and intention to use in the future, in a sample of college students. We hypothesized that compared to baseline, NIPA would result in reduced emotional distress, risky decision-making, cognitive difficulties, and substance use.

## Method

### Design

**Figure 1:**
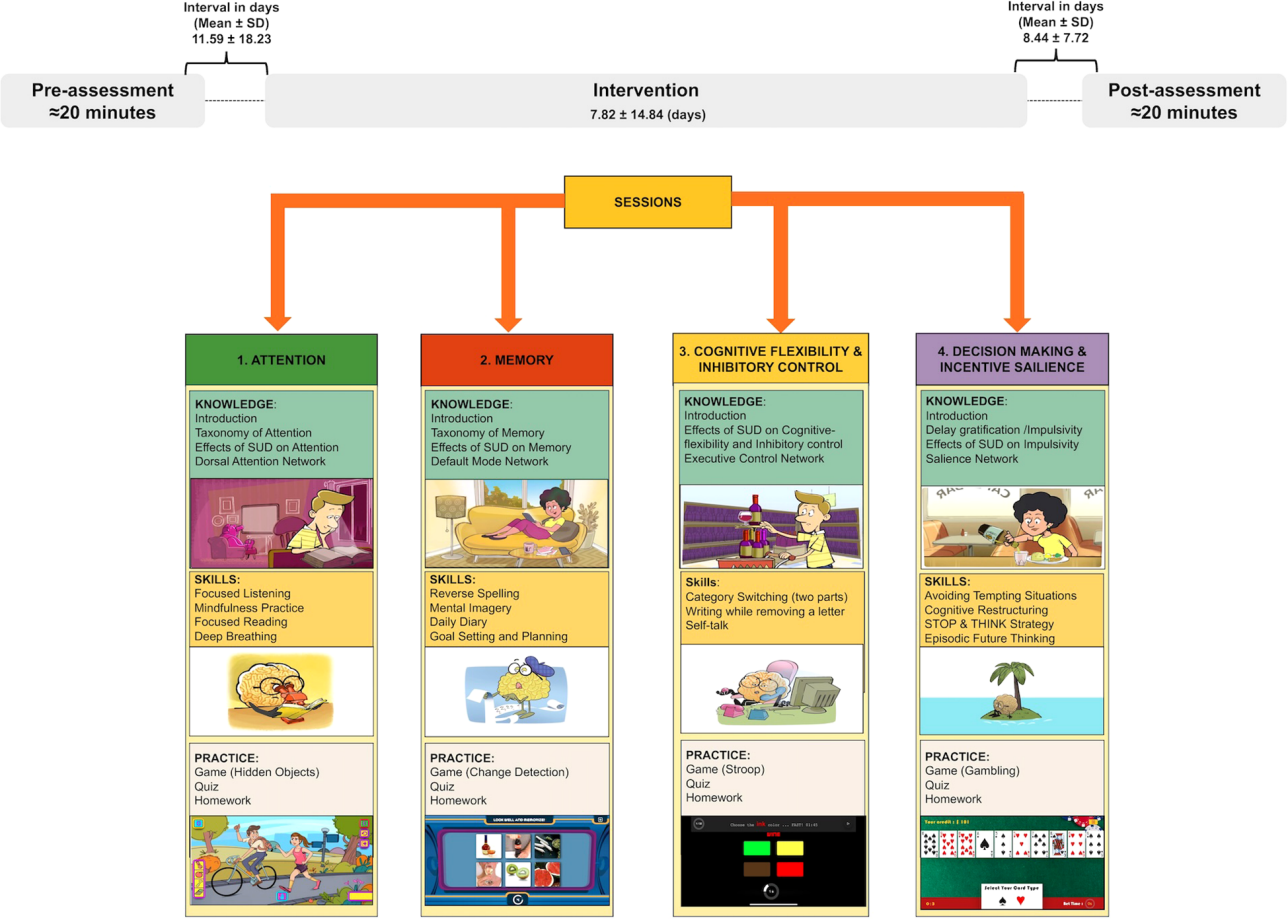
Study design including pre- and post-intervention self-report assessments and four intervention sessions/cognitive modules of the NIPA intervention, including knowledge, skills, and practice sections in four cognitive domains.

A single arm pilot study was conducted in a sample of 68 undergraduate students in 2022-2023. Participants were recruited from an ongoing longitudinal cohort study of college students at a large, urban, mid-Atlantic public university. The study was approved by the university’s institutional review board (HM20018784) and all participants provided informed consent. For a detailed review of study methods see (24 and 22). Participants were invited by email and screened for eligibility. Inclusion criteria included: (1) being an undergraduate student age 18 years or older; (2) having ever used alcohol, and/or tobacco, and/or cannabis, and/or other drugs; and (3) being willing and able to download the app and complete the program. Eligible participants were asked to complete a set of self-report assessments before and after the intervention sessions. Study data were collected and managed using REDCap electronic data capture tools (**Figure 1**) (25, 26). Participants were compensated with Amazon gift cards at study completion.

### Intervention

The “Neuroscience-Informed Psychoeducation for Addiction (NIPA)” program is a metacognitive awareness intervention designed to promote resilience in the face of emotional triggers, particularly those related to drugs and alcohol. NIPA aims to enhance adolescents’ awareness and knowledge about the effects of drugs and alcohol on brain and cognitive function. It incorporates a series of trainings and modified game-based neurocognitive tasks (i.e., embedded figure test (27), change detection task (28), Stroop task (29), Iowa Gambling Task (30)) delivered through engaging media such as cartoons, animations, and videos. NIPA comprises four 20-minute long sessions, each focusing on specific cognitive function typically affected by addiction: (1) Attention; (2) Memory; (3) Cognitive flexibility/Inhibitory Control; and (4) Decision-making/Incentive Salience. Each session follows the same structure. Each session begins with a short video clip describing a specific cognitive problem (e.g., memory difficulties in recalling past information or imagining future events). This is followed by a few multiple-choice questions asking individuals if they have experienced similar problems in their own lives. Participants then engage in a two-level (level 1-2) brain game designed to engage the cognitive functions discussed (e.g., picture memory puzzle). These initial levels of the game are relatively easy, while the next two levels increase in difficulty. Between levels, individuals learn how the game relates to the cognitive functions being discussed and how these functions are engaged in daily life activities. After completing the next two levels of the game (levels 3-4), participants are introduced to the brain networks involved in the targeted cognitive function. They view brain images highlighting the relevant regions (e.g., the Default Mode Network for memory function). Later in the session, threats and harmful effects of drugs and alcohol use (e.g., drug-dependent learning) are explained through a concrete, narrative-driven animated video enriched with scientific evidence. In the final section of the session, participants are trained with a set of cognitive strategies designed to improve the cognitive function discussed in the session (e.g., practicing mental imagery). These strategies and brain-based games are adapted from neuropsychological studies and evidence-based practices in cognitive training. Each session concludes with a wrap-up section and a mini-quiz to assess participants’ learning. The wrap-up highlights the session’s key points, while the quiz consists of 4 multiple-choice questions. Participants receive immediate feedback about their scores on the games and quizzes upon completing the session, enhancing their learning experience. Feedback is also provided after completing each game level, including reaction time, number of incorrect responses, and total score. For the quiz, participants are immediately shown the correct answers. For a detailed description of the program, see File_Sup 1, Rezapour et al. (22).

### Pre- and post-intervention assessments

- *Barkley Deficits in Executive Function Scale (BDEFS)-Short Form*: This scale measures self-reported deficits in executive functioning as they manifest in daily life using a four-point Likert scale ranging from “never or rarely” to “very often” (31). Scores are summed to calculate the total score, with higher scores indicating greater deficits in executive function.
- *Mindful Attention Awareness Scale (MAAS)*: The MAAS is a 15-item scale developed to evaluate open awareness, a core characteristic of dispositional mindfulness (e.g., “I rush through activities without being really attentive to them”) (32). Each item is scored on a 6-point Likert scale ranging from 1 (almost always) to 6 (almost never). The scores are summed to calculate the MAAS total score, with higher totals reflecting greater levels of mindfulness.
- *Monetary Choice Questionnaire (MCQ)*: The MCQ is a 27-item questionnaire measuring delay discounting (33). It consists of dichotomous choices between a smaller, immediate monetary reward (e.g., $25 today) and a larger, delayed monetary reward (e.g., $75 in 61 days). Both the magnitude of the rewards and the delay to the larger reward systematically vary across items. The MCQ includes three sets of 9 items, categorized by the magnitude of the delayed reward: small ($25, $30, or $35), medium ($50, $55, or $60), and large ($75, $80, or $85). The test provides three separate values of the discounting rate parameter *k* (for small, medium, and large rewards), as well as an overall *k* value.
- *Probability Discounting Questionnaire (PDQ)*: PDQ measures a different type of discounting, more directly related to risk preferences, in contrast to the time preferences indexed by delay discounting (34). It is comprised of three parts, each containing 10 hypothetical questions involving choices between different monetary amounts delivered probabilistically. Each question involves a smaller but certain amount of money, pitted against a larger amount of money delivered probabilistically. The probabilities of obtaining the larger outcome ranged from 10% to 83%; 18% to 91%; and 40% to 97%, for parts 1, 2, and 3, respectively. For example: “Which would you prefer: $40 for sure, or a 2-in-11 chance (18%) of winning $100?” Within each part, the monetary amounts of the smaller certain and larger probabilistic alternatives were held constant. We calculated three separate values of the discounting rate parameter *h* (for small, medium, and large probabilities), with lower *h* values indicating greater preference for probabilistic rather than certain rewards (i.e. higher risk taking).
- *PROMIS*: To measure emotional distress we used the two brief scales from the Patient-Reported Outcomes Measurement Information System (PROMIS; 35). The PROMIS Anxiety Scale is a 4-item self-report measure which assesses anxiety symptoms (e.g., hyperarousal) experienced in the past 7 days. The PROMIS Depression Scale is a 4-item self-report measure which assesses four domains of depression: negative mood, views of self, social cognition, and decreased positive affect and engagement. Subjects rate the frequency of experiencing each symptom on a 5-point Likert-type scale, from 1 (never) to 5 (always).
- *Alcohol use (past 30 days)*: Alcohol use was measured using the frequency and quantity items from the Alcohol Use Disorders Identification Test (AUDIT) adapted to the last 30 days. For frequency, participants were asked on how many days they consumed one or more alcoholic drinks. For quantity, they were asked how many drinks they typically consumed on a drinking day. Participants were also asked about binge drinking, defined as consuming four or more drinks in a row for females and five or more drinks for males. Responses were recorded using a Likert scale.
- *Drinking Motives*: We measured motives for drinking using a 4-item subset from the Drinking Motives Questionnaire (36). One item for each of 4 subscales was included: Social Motives, Conformity Motives, Coping Motives, and Enhancement Motives. Response options are on a Likert-type scale ranging from “strongly agree” to “strongly disagree.”
- *Intentions for alcohol use over the next month* were assessed with 2 items: “How frequently do you intend to drink alcohol during the next month?” and “How frequently do you intend to get drunk during the next month?”, rated on a four-point scale (0 = I don’t drink, 1 = less than last month, 2 = similar to last month, 3 = more than last month).
- *Nicotine use*: Recent nicotine use was assessed by asking participants on how many days they smoked cigarettes and on how many days they vaped nicotine in the past 30 days. These questions were adapted from the National Survey on Drug Use and Health (NSDUH) (37). Responses were rated on a 7-point Likert scale ranging from 0 to 7, with higher scores indicating more frequent use.
- *Cannabis use*: Recent cannabis use and future intentions to use were evaluated with three questions adapted from the NSDUH (37): (1) the frequency of cannabis use during the last 30 days; (2) the number of days participants vaped THC during the past 30 days; and (3) how often do participants intend to use cannabis in the next month.
- *Cannabis use motives:* Motives to use cannabis were queried using the 4 drinking motives items described earlier, adapted for cannabis.

### Statistical analysis

Descriptive statistics (mean scores and standard deviations) for all emotional and cognitive variables were first calculated and then tested for normality using the Shapiro-Wilk test. As the data did not follow a normal distribution, we used the non-parametric Wilcoxon signed-rank test to compare pre- and post-intervention scores. For substance use behaviors and intentions to use, where baseline scores were generally low, we categorized participants’ responses based on direction of change. Positive change was defined as any indication of reduced intention to use, reduction in use, or maintaining nonuse level. Negative change reflected increased intention to use, increased use, or continued use at the same level as pre-intervention. For example, if a person selected “I didn’t drink in the past 30 days” for the question “*On how many days did you drink one or more drinks of an alcoholic beverage*?” before the intervention and selected the same option after the intervention, this was considered a positive change, as their behavior did not shift toward increased use. However, if they changed their response to “Once or twice,” it was considered a negative change. We used binomial tests to determine whether the proportion of participants indicating positive versus negative change differed significantly from chance. All data analyses were conducted using SPSS version 29.0.2.0, with a statistical significance level set at 5% (p < 0.05).

## Results

### Participant characteristics

Participants included 68 undergraduate students (19.09 ± 0.33 years; 83.8% female) who were invited by email and screened for eligibility (**Table 1**).

**Table 1:** Demographic and substance use characteristics (n=68).

Variables	n (%)
Biological Sex	
Female	57 (83.8%)
Male	11 (16.2%)
Race	
Asian	20 (29.9%)
African American	17 (25.5%)
Hispanic	7 (10.4%)
White	21 (31.3%)
Unknown	2 (3%)
History of Substance Use	
History of alcohol use over the past year (Yes)	38 (55.9%)
Life-time history of nicotine use (Yes)	11 (16.2%)
Life-time history of cannabis use (Yes)	32 (47.1%)
Life-time history of Stimulants^a^ Use (Yes)	3 (1.8%)
Life-time history of Opioids^b^ Use (Yes)	1 (0.6%)
Life-time history of Sedative/Hynotics Use (Yes)	2 (1.2%)
Life-time history of Cocaine Use (Yes)	2 (1.2%)
Life-time history of Hallucinogens Use (Yes)	6 (3.7%)

^a^Stimulants include amphetamines, methamphetamines, speed, crystal meth, Ritalin. ^b^Opioids include heroin, morphine, opium, fentanyl, methadone, buprenorphine, oxycodone/oxycontin, tramadol, codeine.

### Comparisons of pre- to post-intervention assessments

#### Cognitive outcomes

We observed a significant reduction in self-reported deficits in executive functioning on the BDEFS (*p*<0.001). There was also an increase in mindful awareness on the MAAS from pre- to post-intervention (*p*=0.002) (**Table 2**, **Figure 1**). This suggests that after completing the intervention sessions, participants reported fewer daily problems related to executive functioning, while experiencing a higher level of metacognitive awareness and mindfulness. Additionally, post-intervention, there was a significant reduction in delay discounting of large magnitude rewards (large *k*) (*p*=0.035), indicating that participants were more willing to wait for larger delayed rewards rather than choosing smaller immediate ones after the intervention. Also, after completing the intervention, participants showed a significant increase in small probability discounting (small ℎ), indicating increased preference for certainty and greater risk aversion (*p*=0.001).

**Table 2:** Comparison of cognitive and emotional outcomes between pre- and post-intervention (n=68).

Variable	Pre- Mean (SD)	Post- Mean (SD)	Z	P	95% CI for the difference	
					Low	Upper
BDEFS score	37.51 (11.95)	15.40 (11.11)	-7.11	<0.001^*^	0.000	0.006
MAAS score	54.38 (12.12)	57.62 (19.16)	-3.07	0.002^*^	0.000	0.010
DD_Overall *k*	-2.44 (0.76)	-2.33 (0.85)	-1.52	0.127	0.104	0.164
DD_Small *k*	-2.21(0.80)	-2.12 (0.87)	-0.75	0.450	0.442	0.530
DD_Medium *k*	-2.44 (0.78)	-2.32 (0.88)	-1.55	0.120	0.097	0.155
DD_Large *k*	-2.66 (0.72)	-2.50 (0.85)	-2.11	0.035^*^	0.005	0.027
PDQ_Small ℎ	2.21 (3.13)	3.37 (4.2)	-3.177	0.001^*^	0.000	0.006
PDQ_Medium ℎ	1.84 (2.42)	1.98 (2.55)	-1.145	0.252	0.214	0.290
PDQ_Large ℎ	1.66 (2.36)	1.54 (2.89)	-1.36	0.173	0.137	0.203
PROMIS Depression	56.76 (10.34)	54.85 (9.62)	-2.58	0.010^*^	0.000	0.013
PROMIS Anxiety	60.24 (9.50)	58.43 (9.69)	-2.49	0.013^*^	0.002	0.022

BDEFS, Barkley Deficits in Executive Function Scale; MAAS, Mindful Attention Awareness Scale; DD, Delay Discounting; PDQ, Probability Discounting Questionnaire. *p<0.05.

#### Emotional outcomes

Significant changes were observed in both subjective rating of depression (*p*=0.010) and anxiety (*p* =0.013) from pre- to post-intervention. This suggests that receiving the intervention may have decreased emotional distress and improved mental health (**Table 2**, **Figure 1**).

#### Substance use outcomes

**Figure 2:**
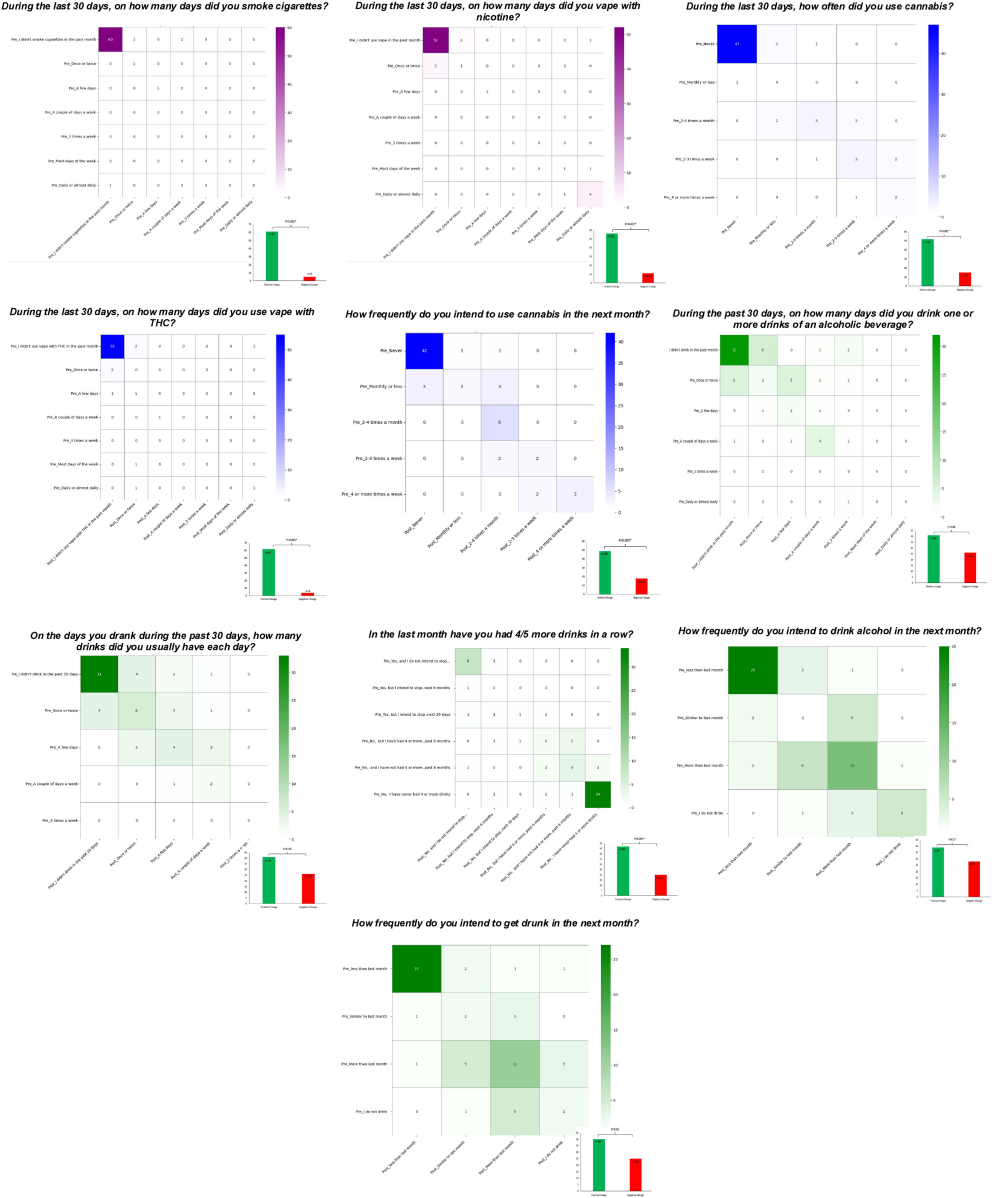
Heat maps of the number of survey participants for each question related to substance use and intentions to use from pre- to post-intervention.

We found that motivation to drink alcohol and use cannabis —including social, conformity, coping, and enhancement motives—remained consistent before and after the intervention (**Table 3**). For substance use variables, where baseline scores were low, we categorized responses based on individual-level positive or negative change from pre- to post-assessment (**Figure 2**). Our findings indicate reduced frequency of cannabis use and intentions to use cannabis, as well as reduced tobacco use following the intervention (*p*<0.001). There was also a significant reduction in binge drinking (*p*=0.01); however, there were no significant changes in intentions to use alcohol and to get intoxicated (*p*>0.05).

**Table 3:** Comparison of pre- and post-intervention scores on alcohol and marijuana use motives.

Items (*n* ^*^)	Pre- Mean (SD)	Post- Mean (SD)	Z	P
Motivation for drinking: It makes social gatherings more fun (*n=63*)	2 (0.93)	2.16 (0.98)	-1.5	0.13
Motivation for drinking: To get in with a group (*n=65*)	3.45 (0.82)	3.59 (0.72)	-1.35	0.17
Motivation for drinking: It helps me when I feel depressed or nervous (*n=63*)	2.84 (0.98)	2.89 (1.07)	-0.47	0.63
Motivation for drinking: It gives me a pleasant feeling (*n=63*)	2.21 (0.90)	2.27 (0.83)	-0.51	0.60
Motivation for using marijuana: It makes social gatherings more fun (*n=59*)	2.87 (1.07)	2.92 (0.97)	-0.66	0.50
Motivation for using marijuana: To get in with a group (*n=58*)	3.53 (0.76)	3.57 (0.78)	-0.53	0.59
Motivation for using marijuana: It helps me when I feel depressed or nervous (*n=59*)	2.86 (1.07)	3.05 (1.01)	-1.64	0.10
Motivation for using marijuana: It gives me a pleasant feeling (*n=59*)	2.60 (1.15)	2.75 (1.14)	-1.12	0.26

^*^n is the number of respondents to each item.

## Discussion

The present study investigated the efficacy of a novel mobile-based intervention using a neuroscience-based psychoeducation approach in a sample of college students with a lifetime history of alcohol, tobacco, cannabis, or other drug use. The program implemented a multimedia approach, using cartoons, animations, and videos to convey educational materials about the effects of drugs and alcohol on brain structure and function, and provided applicable knowledge about cognitive functions to foster individuals’ metacognitive awareness. Our results support the utility of the program for improving neurocognitive function, metacognitive awareness, and mental health, as well as for reducing intentions to use cannabis, actual cannabis and nicotine use, and binge drinking in our sample.

It is essential to highlight the educational content of NIPA when we consider these findings, particularly its focus on cognitive functions and associated brain networks. In each session, participants are introduced to scientific concepts related to specific cognitive functions—including attention, memory, cognitive flexibility, and decision-making—and how impairments in these functions may manifest in everyday life. This information is conveyed using language and vignettes that are familiar and relevant to younger audiences. Each session begins with thought-provoking questions, accompanied by a comic-style animation illustrating a real-world cognitive challenge. This approach is designed to encourage self-reflection and activate metacognitive processes, prompting individuals to consider whether they have encountered similar challenges in their own lives. This engaging introduction primes participants for the subsequent questions, which explore potential causes of these cognitive problems. As a result, the information that follows is more likely to be interpreted as self-relevant, processed attentively, and consolidated as meaningful. Throughout the sessions, participants gain both scientific and practical knowledge related to each cognitive function. They also watch a schematic video depicting specific brain regions and networks involved in these processes. These visual illustrations are useful heuristics, making it easier for participants to recall session content later (38). Moreover, individuals are continuously encouraged to appraise their cognitive strengths and weaknesses, enhancing their motivation to learn and apply the strategies presented in each session to improve these functions. Given the emphasis on metacognitive awareness in the educational content, it is not surprising that after completing the intervention, participants reported fewer problems with executive cognitive functions (as measured by the BDEFS) and higher metacognitive awareness (as measured by the MAAS).

A particularly interesting finding was the impact of NIPA on decision-making, especially in situations involving risk and uncertainty, as assessed by the delay discounting and probability discounting questionnaires. We found that following the intervention, participants showed a significantly greater preference for waiting for larger delayed rewards over opting for smaller, immediate rewards. Additionally, they became more sensitive to uncertainty and showed greater risk aversion. These effects may have been due to the exclusive focus on practicing mindfulness, goal setting, planning, mental imagery, and future thinking over the four sessions. These results extend previous studies showing the effect of mindfulness training (39), imagining future events (40), and goal management training (41) on decision making.

Another key finding was related to emotional outcomes, specifically symptoms of depression and anxiety. After completing the four intervention sessions, participants reported improved emotional well-being. One possible explanation is that neuroscience-informed psychoeducation enhances self-awareness and fosters a sense of voluntary control over emotional processes. These preliminary findings align with theoretical models suggesting that metacognition plays a role in emotion regulation strategies (42). Notably, to our knowledge, this is the first study to demonstrate that a neuroscience-informed psychoeducational program can influence psychological distress and mental health. Previous studies, such as those by Debenham (20, 43) and Meredith (21) have focused primarily on drug use and drug literacy rather than emotional outcomes.

Finally, our program was effective in reducing binge drinking, intentions to use cannabis, and actual cannabis and nicotine use. After completing the intervention, a significantly greater number of participants reported positive changes in substance use, indicating reduced intention to use or reduced actual use. However, no significant effects were observed for other measures related to reductions in alcohol use, intentions or motivations to drink alcohol. The most likely explanation for these non-significant findings may be the lesser emphasis on alcohol compared to other drugs in the educational content of NIPA.

Overall, rather than explaining drug addiction in a fearful or stigmatizing manner, NIPA provides individuals with an opportunity to engage in self-related processes, enabling them to allocate their cognitive and emotional resources toward adjusting their thoughts, emotions, and behaviors, thereby increasing their self-awareness and metacognitive awareness. The effects of the app on cognitive, emotional, and substance use outcomes may be explained by increases in metacognitive awareness This study is among the first to develop neuroscience-informed psychoeducation specifically focused on the neurocognitive aspects of substance use and their influence on daily living, integrating both theoretical and practical knowledge through multimedia approach, including cartoons, animations, videos, and games. However, several limitations should be considered.

First, our sample was quite small and consisted of mostly female students from a single university, which may limit the generalizability of the results. Greater sample diversity in future studies would allow the investigation of gender effects in response to psychoeducation, which could later inform the personalization of educational content. Second, the prevalence of substance use was relatively low in our sample, and most participants maintained their non-using status after completing the intervention. Although we considered this stability as a positive change, to gain better insight into the program’s effectiveness, future studies would benefit from including students who actively misuse substances. Third, our study lacked an active control group that received an alternative form of psychoeducation (i.e., conventional type of educational program about substance use disorders), which would allow for a comparison of the added value of the neuroscience-informed approach for improving metacognitive awareness and intentions for future substance use. Moreover, to assess the transfer and durability of potential changes over time, future studies should include follow-up assessments. Fifth, our study assessed only the immediate effects of the intervention, and the time spent to complete the four training sessions varied among participants. In the future, longer follow-up periods and a unified training schedule should be implemented to better evaluate the long-term impact of the intervention. Last but not least, we relied on self-report measures, which may not fully capture key outcomes such as cognitive deficits, intentions, and actual substance use. To address these limitations, future studies would benefit from incorporating a combination of self-report, neuropsychological and behavioral assessments, as well as more validated ecological momentary assessment (EMA) methods, which better reflect real-world outcomes and reduce biases such as self-confirmation and memory recall errors.

To address these gaps, future studies should improve both the app and the study methodology. Regarding the app, we plan to update the educational content to include specific drug related education about commonly used drugs (e.g. alcohol, opioids, stimulants, cannabis, nicotine), while correcting common myths that are widely believed among adolescents. To enhance user engagement, we will integrate interactive homework exercises for each session. The homework will allow users to practice the strategies they have learned, helping them transfer what they have learned into real-life situations. Moreover, based on the feedback from our pilot study (22) that the brain-based strategies were the least favorite part of the sessions, we will revise this section to make it more engaging and user-friendly. Another modification planned for the next version is to personalize the feedback to participants based on their cognitive profiles, generated from their performance on the cognitive games and assessments within the application. To strengthen methodological rigor, our next step will involve using the updated version of the application in a randomized clinical trial with a control group across a few universities. The program’s efficacy will be evaluated using a comprehensive set of self-report measures and neuropsychological assessments administered at pre-intervention, post-intervention, and follow-up to monitor potential changes over time in a sample of male and female students.

## Conclusion

Our preliminary results hold promise for the efficacy of educational programs inspired by advances in neuroscience for reshaping substance use intentions, attitudes, and behaviors of young adults. Interventions such as NIPA could help correct individuals’ beliefs about the harmful effects of drugs, while increasing their sense of self-agency to control their cognitive and emotional processes, and, consequently, their behaviors.

## Data Availability

The raw data supporting the conclusions of this article will be made available by the authors, without undue reservation.
