# Supplementary material for: Efficacy of a neuroscience informed psychoeducation intervention on cognitive, emotional, and substance use outcomes in college students: a pilot study
Source: Front Psychiatry. 2025 Sep 18;16:1655909. doi: 10.3389/fpsyt.2025.1655909 (PMC12488669; doi:10.3389/fpsyt.2025.1655909)
Supplement: Supplementary file 1 [file Table1.docx]

Supplementary File

Description of the program

Once individuals install the NIPA app on their mobile phones, the first session is unlocked, providing access to its full content. Throughout all the sessions, we use different comic characters, including 'Mr. Brain' to narrate complex brain-based concepts and add a sense of humor to make the content more engaging. All four sessions follow a similar structure, each taking approximately 15–20 minutes. Each session includes the following sections:

- Introduction (Knowledge): Each session begins with an animation depicting a specific cognitive problem (Session 1: Attention and Concentration; Session 2: Memory; Session 3: Cognitive Flexibility and Response Inhibition; and Session 4: Impulsivity and Decision-Making). Subsequently, individuals are asked about their real-life experiences with the cognitive problem(s). For example, in the first session, we explain how attention problems can manifest in daily life activities, such as studying, and how they make it challenging to filter out distractions such as environmental noise.

- Games (Practice): Following the introduction and exploration of specific cognitive functions and difficulties, individuals play the first two levels of a game (levels 1-2), which engages the specific cognitive processes reviewed in that session. The games are designed to raise individuals' awareness of how they use specific cognitive functions to solve game-based scenarios. For instance, in the second session after watching an animation illustrating how memory difficulties can interfere with recalling past memories, individuals play a picture memory game, which engages their recognition memory to recognize pictures they have just seen. After the neuroscience-informed psychoeducational section (described below), individuals repeat the game with an increased level of difficulty (levels 3-4), this time with greater awareness about the specific cognitive processes involved.

- Neuroscience-informed psychoeducation (Knowledge): This animated section aligns with the previous ones and explains specific cognitive functions implicated in addiction and their underlying brain networks. These networks include the Attention Network (AN), responsible for attention; the Default Mode Network (DMN), involved in memory and interoceptive processes; the Executive Control Network (ECN), associated with inhibitory control and cognitive flexibility; and the Salience Network (SN), which assigns salience to emotional stimuli. For example, in the third session participants learn about flexibility and inhibition of automatic behavioral responses as well as the underlying neural network, such as the ECN which includes the dorsolateral prefrontal cortex (dlPFC), the posterior parietal cortex, and the dorsal anterior cingulate cortex. Moreover, immediately after playing the second round of games, individuals are provided with additional scientific evidence on how different brain regions within a network are activated to invoke a cognitive function. In this section, individuals also learn about specific threats to brain functions as a result of using substances and other stressors. For example, in the third session, the concept of perseveration is introduced as a result of the disruption of the normal functioning of flexibility. This section is presented through engaging cartoons and animations.

- Brain training Strategies (Skills): The final section of each session is dedicated to providing four specific cognitive training strategies to boost the specific cognitive functions reviewed in the session, aiming to improve individuals' resilience when exposed to drugs and other stressors. Each strategy is accompanied by an exercise where individuals are required to apply the strategy they have learned. For example, in the fourth session, Cognitive Reappraisal, Stop and Think, and Episodic Future thinking skills are learned and practiced through exercises.

- Wrap-up (Practice): Once individuals complete each session, they are provided with session highlights and are then directed to a multiple-choice quiz, consisting of 4-5 questions designed to improve the learning experience. Immediately after completing the quiz, they receive feedback with their scores on the games and quizzes.
